# Pars Plana Vitrectomy Alone or Combined with Phacoemulsification to Treat Rhegmatogenous Retinal Detachment: A Systematic Review of the Recent Literature

**DOI:** 10.3390/jcm12155021

**Published:** 2023-07-30

**Authors:** Carlo Bellucci, Alessandra Romano, Francesca Ramanzini, Salvatore Antonio Tedesco, Stefano Gandolfi, Paolo Mora

**Affiliations:** Ophthalmology Unit, University Hospital of Parma, 43126 Parma, Italy

**Keywords:** pars plana vitrectomy, rhegmatogenous retinal detachment, lens-sparing vitrectomy, cataract surgery, phacovitrectomy

## Abstract

Pars plana vitrectomy is today a common first-line procedure for treatment of rhegmatogenous retinal detachment (RRD). Removal or preservation of the natural lens at the time of vitrectomy is associated with both advantages and disadvantages. The combination of cataract extraction (i.e., phacoemulsification) with pars plana vitrectomy (PPVc) enhances visualization of the peripheral retina and the surgical management of the vitreous base. However, PPVc prolongs the surgical time and is associated with iatrogenic loss of the accommodation function in younger patients, possible postoperative anisometropia, and unexpected refractive results. Performance of pars plana vitrectomy alone (PPVa) requires good technical skills to minimize the risk of lens damage, and quickens cataract development. We retrieved all recent papers that directly compared PPVc and PPVa using parameters that we consider essential when choosing between the two procedures (the success rate of anatomical RRD repair, postoperative refractive error, intra- and postoperative complications, and costs). PPVa and PPVc were generally comparable in terms of RRD anatomical repair. PPVc was associated with fewer intraoperative, but more postoperative, complications. Macula-off RRD PPVc treatment was often associated with undesirable myopic refractive error. PPVa followed by phacoemulsification was the most expensive procedure.

## 1. Introduction

Rhegmatogenous retinal detachment (RRD) is a major vision-threatening disease with an incidence ranging from 6.3 to 17.9 per 100,000 people per year. RRD develops after retinal tears caused by trauma, or related to retinal structural anomalies, pathological myopia, complicated cataract surgery, or posterior vitreous detachment. Through these tears, vitreous chamber fluid accumulates between the neurosensory retina and the retinal pigment epithelium. When the retina separates from the pigment epithelium, surgical management is required [1,2,3,4]. Although RRD is more common in adult myopic and/or pseudo-phakic patients, retinal tears may also occur in younger patients, especially after trauma or associated with hereditary collagen anomalies [5,6,7]. There are currently three surgical approaches for RRD management: scleral buckling, pneumatic retinopexy, and pars plana vitrectomy (PPV). Buckling is the oldest method. In this method, silicone bands are placed outside the ocular globe and sutured to the sclera to relieve vitreal traction and approximate the pigment epithelium to the retinal tears. This procedure may be combined with retinopexy. Pneumatic retinopexy is usually employed to treat only RRD cases with few, small retinal tears in the superior quadrants of the eye. Cryo-coagulation is applied onto the margins of the tears followed by the intraocular injection of an air/gas bubble. The appropriate postoperative head positioning is essential to allow the bubble to tamponade the retinal tear. The combination of PPV with an encircling band is also worth mentioning, particularly for cases at high risk for proliferative vitreoretinopathy.

Currently, the primary anatomical success rate of PPV using 23/25/27-gauge instruments is 80% [8,9,10]. The vitreous is fragmented and aspirated, thus removing the retinal traction and flattening the detached retina, followed by tamponade with air, gas or silicone oil. Despite the success of repair, a frequent side-effect of PPV performed on a phakic eye is progressive cataract development and thus a rapid need for cataract surgery. This is technically more challenging than usual because the vitreous support is lacking [11,12]. A combination of phacoemulsification with PPV affords certain advantages, including better visualization during retinal surgery and wider access to the vitreous base. This allows more complete vitreous shaving and facilitates intraoperative laser treatment. Tamponade filling is also more extensive, thus reducing the risk of RRD recurrence. However, the combined procedure also has certain disadvantages, such as a longer surgery time with possible additional complications (e.g., corneal edema), increased risk for postoperative refractive errors (when the macula is detached, the axial length (AL) measurement may be erroneously short if particular caution is not taken), and postoperative anisometropia in myopic eyes. Removal of the natural lens is also associated with complete loss of any residual accommodation function [13,14,15,16,17,18,19,20,21,22,23,24].

Both phacoemulsification combined with PPV (PPVc) and PPV alone (PPVa) are associated with some advantages and disadvantages. Surgeons usually select treatment by reference to the patient characteristics and their personal experience. A recent meta-analysis reported that the two techniques yield statistically comparable results, with the exception that PPVa is associated with slightly better final visual acuity [25]. The present review complements the cited study by considering additional publications and peculiar clinical and technical parameters.

## 2. Materials and Methods

PubMed, EMBASE, and the Cochrane library were searched between January and April 2023 for papers comparing PPV alone, and combined with phacoemulsification, as RRD treatments published from January 2010 to March 2023. We did not consider earlier papers because PPV instrumentation has evolved consistently over the last decade. The following keywords were used separately and in combination: “Vitrectomy”, “Pars plana vitrectomy”, “PPV”, “Lens-sparing vitrectomy”, “Vitrectomy with cataract surgery”, “Phacovitrectomy”, “Vitrectomy with phacoemulsification”, “Combined vitreoretinal surgery”, “Rhegmatogenous retinal detachment”, and “Retinal detachment.”. Papers addressing at least one of the following issues were included: rate of RRD anatomical repair, postoperative refraction, rate(s) of intra and/or postoperative complications, and costs.

Preprints, papers not in English, non-peer-reviewed studies, and papers using PPV to treat conditions other than RRD were excluded.

## 3. Results

The search yielded 3319 citations, reduced to 13 after removal of duplications and irrelevant works, with a total of 7379 eyes considered. The characteristics of the included studies are listed in Table 1.

For each of the four chosen relevant issues, cited studies are presented in chronological order and the values of statistical significance (p) are detailed in the respective tables.

### 3.1. Efficacy of RRD Repair

Data on primary anatomical healing rate after RRD was treated via PPVa and PPVc are given in Table 2.

The earliest paper that we reviewed was that of Caiado et al. [19] This retrospective study included 97 RRD eyes classified by lens status (phakic or pseudo-phakic), the surgical technique used (23-gauge PPVc or PPVa), and the tamponade agent (long-lasting gas or silicone oil); the minimum follow-up was 12 months. The primary PPV success rate for pseudo-phakic eyes, using either a gas or silicone oil tamponade, was higher than that for phakic eyes, suggesting that the combination of phacoemulsification with PPV was valuable in terms of RRD anatomical repair.

Loukovaara et al. [17] retrospectively assessed 1690 consecutive RRD patients, of whom 1564 (92.5%) underwent PPVa and only 126 (7.5%) received PPVc. The primary endpoint was the reoperation rate to 1 year. The reported RRD repair efficacy rate was 90.5% for PPVa and 81% for PPVc but the authors did not mention the criteria used to address the patients to PPVa (the large majority) or to PPVc.

Guber et al. [21] retrospectively studied 1017 RRD eyes, of which 516 (50.7%) underwent PPVc and 501 (49.3%) received PPVa. No significant between-group differences in the redetachment rate were noted, this being 9.6% in the PPVc group and 10.7% in the PPVa group. However, the details of postoperative follow-up were not described.

More recently, Helmy et al. [28] published a comparative, prospective randomized study that compared two groups each of 20 patients, of which the first underwent primary PPVa with a silicone oil tamponade followed 3 months later by silicone oil removal in combination with cataract surgery. The second group underwent PPVc followed by silicone oil removal only 3 months later. No patient in both groups had recurrent RRD under silicone oil. One eye of the first group had retinal re-detachment 1 month after the second procedure (phaco-silicone removal).

The retrospective, comparative case series of Tan et al. [22] included 127 phakic RRD eyes treated via PPVc (mean follow-up 42.6 ± 14.3 months) and 139 such eyes treated with PPVa (mean follow-up 41.6 ± 15.8 months). The primary reattachment rates were 84.3% for PPVc and 89.2% for PPVa. 

Mora et al. [4] conducted a prospective comparative trial with 6 months of follow-up. Fifty-nine eyes of 59 patients were consecutively enrolled for the study and randomly allocated to PPVa (29 eyes) or PPVc (30 eyes). The groups were comparable in terms of demographics, extension and locations of the retinal breaks, and the tamponade used (gas in all the cases). The log-rank test revealed that the relative risk of RRD recurrence was 3.22 times higher in PPVa group than in PPVc group, but the difference was not statistically significant (*p* = 0.3). The authors postulated that the higher risk could be related to a non-near complete vitreous removal and the possible missing of some very peripheral adjunctive tears when the lens has to be preserved.

During a retrospective review of the medical records of 193 patients with primary RRD, Kim et al. [23] included those who underwent either PPVa (111) or PPVc (82). The mean follow-up period was 26.3 months. Lens-sparing procedures were performed on 60 eyes (58.8%) by experienced surgeons and on 51 eyes (56.0%) by surgical fellows; PPVc was performed on 42 eyes (41.2%) by experienced surgeons and on 40 eyes (44.0%) by fellows. In the PPVa group, the RRD repair efficacies did not significantly differ (91.7% for experienced surgeons and 94.1% for fellows). In the PPVc group, the primary success rate was higher when surgery was performed by senior surgeons (97.6% vs. 85% for fellows). This difference was termed “marginally significant.”

Radek et al. [30] retrospectively analyzed 5502 eyes with uncomplicated primary RRD operated upon by 13 vitreoretinal surgeons with different levels of surgical experience in a single center over a 15-year period. Of 2614 phakic eyes treated via PPV, 2163 (82.7%) underwent PPVc and the rest received PPVa. The RRD anatomical repair rate was better in the group that underwent combined surgery (93%) than in the PPV-alone group (88.7%). Relating to this evidence, the authors commented that the difference was eventually very small and possibly influenced by some issues such as the lack of randomization, the fact that the two groups differed in several aspects, first of all size, and that the PPVc patients were older and operated upon later in the observation period of the study.

### 3.2. Postoperative Refraction

Postoperative refraction data after both PPVa and PPVc are listed in Table 3.

The earliest relevant paper was Kim et al. [27] The authors retrospectively compared a “combined procedure group” (cataract surgery immediately followed by PPV, 38 eyes) to a “delayed group” (cataract surgery performed with an average delay of 6 months after PPVa, 25 eyes). Gas tamponade was used in all the included patients. AL and keratometry were measured by immersion A-scan ultrasound (patient in supine position) and by auto refracto-keratometer, respectively. For 54 of the 63 cases, intraocular lens (IOL) Master Biometry (Carl Zeiss Meditec AG, Germany) was also performed, with patients upright. The SRK/t formula was used for IOL power calculation (eyes which had undergone prior refractive surgery were excluded) and the final refractive error evaluated at least 1 month after cataract surgery. A significant myopic shift of −0.40 ± 1.07 D was found in the PPVc group; the mean refractive error was only 0.07 ± 0.56 D in the PPVa group. The study did not specify if ultrasound measurements were taken automatically, or a manual caliper correction was admitted.

Tan et al. [22] measured kerato-metric values using an auto refracto-keratometer and derived the AL via optical biometry (IOL Master). The AL was also checked using immersion A-scan ultrasonication in patients with macula-off RRD or dense optic media. If the AL measurement was significantly shorter than that of the fellow eye by both methods, and no preexisting anisometropia was apparent, the final refractive target was determined using the AL of the fellow eye. The SRK/t formula was used in all cases (prior refractive surgery was among the exclusion criteria). In line with the findings of other studies, the PPVc group exhibited a greater myopic refractive error than did the delayed group (−0.32 ± 1.28 D vs. 0.16 ± 1.53 D) but the difference was significant only for patients with macula-off RRD. As for the above discussed study, methods did not detail if the manual correction of the ultrasounds calipers was adopted.

Mora et al. [4] used optical biometry (IOL Master 500) to derive the AL of cases with macula-on RRD, derived kerato-metric values, and measured anterior chamber depths. Combined vector-A/B-scan ultrasound biometry was employed to measure AL in patients with macula-off RRD. The IOL power was calculated using the SRK/t formula for all eyes except those that had undergone prior refractive surgery, for which the Barrett true-K formula was employed. Refractive outcomes were not reported. The visual outcome was somewhat better in the PPVa group but statistical significance was not attained. At the 6-month follow up, only 16/30 eyes (53%) in the PPVc group had attained the preoperative refractive target (previously unpublished data).

In a retrospective comparative study of 154 eyes with RRD, Moussa et al. [29] evaluated the accuracies of optical biometry, swept-source optical coherence tomography, and contact ultrasound biometry in determining the refractive outcome. The authors also considered the effect of macular status. Three groups were defined: phakic patients with RRD treated via PPVc (70 eyes) (group 1), RRD patients treated via PPVa (41 eyes) (group 2), and matched patients without RDD undergoing cataract surgery (controls) (43 eyes) (group 3). An IOL Master 700 was used to obtain optical biometric measurements, but immersion ultrasonography was performed when IOL Master data were considered unreliable, thus for cases with macula-off RRD. The SRK/t formula was used when the AL was >22 mm and the Hoffer Q formula when the AL was <22 mm. Group 1 evidenced an overall myopic shift of −0.08 ± 0.92 D and group 2 a shift of −0.07 ± 0.56 D, not significantly different than that of the control group. However, a wider myopic shift and a significantly increased mean absolute refractive error affected a subgroup including the macula-off RRD eyes, in which the IOL power calculation was obtained by ultrasound. The macula-off eyes in the group 1 had significantly higher mean absolute error when compared to control eyes (*p* < 0.001), while the difference between the macula-off eyes in group 2 and the control group was not significant.

Kim et al. [23] used optical (IOL Master) and ultrasound biometry to assess the refractive outcomes of two groups but did not specify how they chose between the two measurement methods. In addition, the formula employed for IOL power calculation was not mentioned. A combined group evidenced a significant postoperative myopic shift of −0.58 ± 0.97 D compared to a delayed cataract group (−0.18 ± 0.84 D).

We did not discuss in this paragraph the study of Helmy et al. [28] because it mentioned the optical biometer as the sole instrument to measure AL in all the considered eyes.

### 3.3. Intra and/or Postoperative Complications

We do not discuss RRD redetachment rates; these have been reviewed previously.

Lee et al. [18] retrospectively but directly compared the intraoperative complications during phacoemulsification of patients who underwent cataract surgery after PPV (54 patients) and those who received PPVc (311). The main indications for PPV were RRD and vitreous hemorrhage for diabetic retinopathy. A control group of 334 patients underwent cataract surgery alone during the same period. The most common complication during cataract surgery was posterior capsule rupture, which was higher in the sequential (six eyes, 11.4%) than the combined (14 eyes, 4.5%) and control (eight eyes, 2.4%) groups. Lens dislocation and iris trauma were rare and the rates did not significantly differ among the groups.

Erçalık et al. [20] reviewed the records of 834 patients who received PPV, mainly for RRD, proliferative diabetic retinopathy, and epiretinal membrane. 376 patients underwent cataract surgery after PPV (the sequential group) and 458 received PPVc. The principal intraoperative complication was posterior capsule rupture and was significantly more common in the sequential than the combined group. Zonular dehiscence was also significantly more common in the former group. Other complications included iris trauma, lens drop, and complicated capsulo-rhexis, all of which were somewhat more common in the sequential group, but statistical significance was not attained. In terms of early postoperative complications, PPVc eyes exhibited a significant rise in intraocular pressure (IOP), fibrinous exudations with formation of pupillary synechiae, posterior capsule opacification, and IOL dislocation. The incidence of endophthalmitis was comparable between the two groups.

Loukovaara et al. [17] reported more postoperative complications that required additional surgery in a PPVc than a PPVa group. The principal complications in both groups were a secondary epiretinal membrane, a secondary macular hole, vitreous hemorrhage, mechanical IOL complications, secondary glaucoma, complicated cataracts, and suspected endophthalmitis.

Helmy et al. [28] did not find statistically significant differences in intraoperative complication rates among two groups studied. However, the PPVc group evidenced a higher rate of early postoperative complications, including an increase in IOP associated with a need for anti-glaucoma drops (13 patients, 65%), corneal edema (six patients, 30%), and anterior chamber reactions (14 patients, 70%). Moreover, 11 patients (55%) in the combined group experienced emulsification of silicone oil in the anterior chamber compared to only one patient (5%) in the PPVa group.

In terms of peri- and postoperative complications, Tan et al. [22] did not find any significant difference between two groups studied. One case of posterior capsule rupture occurred in each group. In terms of postoperative complications, the rates of epiretinal membrane formation and macular edema were similar in the two groups. Combined cataract extraction was associated with more rapid visual recovery. A total of 109 eyes (78.4%) in the PPVa group required cataract extraction during the follow-up period (mean delay of 8.0 ± 7.5 months, from 1 to 41 months).

Mora et al. [4] found no difference between two groups studied. The cataract status of the PPVa group was prospectively followed-up until cataract surgery was indicated, at a median of 14.5 months from PPV.

Kim et al. [23] reported one case (2.4%) of zonular dialysis during a combined procedure performed by an experienced surgeon and four cases (10%) of posterior capsular rupture during combined procedures performed by surgical fellows. Of 60 patients in the PPVa group treated by experienced surgeons, 32 (53.3%) developed cataracts and underwent subsequent surgery by the same surgeons, of whom one required additional scleral fixation surgery because of IOL subluxation. Of 51 patients in the PPVa group operated upon by fellows, 31 (60.8%) underwent cataract surgery, posterior capsular ruptures occurred in five cases (16.1%), IOL subluxation was observed in two cases (6.5%), and one patient (3.2%) developed intraoperative zonular dialysis. In patients treated by both experienced surgeons and fellows, epiretinal membranes were significantly more common after PPVc (23 patients, 28%) than PPVa (9 patients, 8.1%). Cystoid macular edema developed in 10 patients of the PPVc group (12.2%) but no significant between-group differences were apparent in terms of macular hole formation. 

### 3.4. Costs 

The only paper to compare costs was Seider et al. [26]. The total costs of PPVc and PPVa with subsequent phacoemulsification were derived. These included the fees of surgeons, ambulatory surgical centers, and anesthesiologists. The sequential procedure cost $4680.86 and the combined procedure $3729.88, thus 20.3% less.

## 4. Discussion

Once PPV is chosen to manage RRD in a phakic eye, safety and efficacy rates are key to orient the attitude toward the natural lens. The best repair results are associated with maximal visualization of the posterior surgical planes, i.e., when mydriasis is durable and consistent and the cornea, lens, and vitreous-capsule interface are clear. Any permanent or transitory opacity of these structures compromises various critical PPV maneuvers. Opacities may hide small retinal breaks that may thus remain untreated. They could compromise the full release of vitreous traction in the peripheral retina, render endo-laser treatment challenging, and complicate retinal drying during tamponade exchange. Although PPVa for eyes with clear lenses or mild cataracts is effective and rapid, the well-known risk of subsequent cataract formation and the difficulties associated with phacoemulsification without vitreous support must be considered. Several of the studies that have compared PPVc and PPVa have found that RRD anatomical repair efficacy is statistically comparable if phacoemulsification is performed either at the time of PPV or later [4,19,21,22,28]. One study reported little better anatomical repair results after PPVc [30]. However, that retrospective study evaluated many more PPVc than PPVa patients. In contrast, Loukovaara et al. [17] reported a lower anatomical repair rate of RRD eyes after the combined procedure. However, again, that was a retrospective study in which the numbers in the two groups were not well-balanced. Kim et al. [23] reported that PPVc performed by surgical fellows was associated with marginally poorer results than PPVc performed by experienced surgeons, suggesting that the decision to combine procedures should consider the surgeon’s skill. Longer and more stressful surgery can trigger optical media reactions including corneal edema and myosis.

The refractive outcomes after PPVc and PPVa have also been studied but the methods used to perform preoperative ocular biometry were often described only briefly and differed among the studies [4,22,23,27,28,29]. Visual improvement after RRD cannot be guaranteed, especially when the macula is involved in the retinal detachment. However, the accuracy and appropriateness of ocular biometry is critical when PPVc is planned. Optical or ultrasound biometry can be used to assess the AL, which is the most challenging parameter to obtain in RRD eyes for IOL power calculation. Optically, AL is measured via noncontact, partial, coherence laser interferometry or swept-source optical coherence tomography that consider the retinal pigment epithelium signal. Ultra-sonographically, AL is measured via A-scan ultrasound that considers the internal limiting membrane signal. A detached macula may interfere with such measurements, yielding an erroneously short AL, associated with a possible myopic refractive error after cataract extraction [31]. Postoperative, myopic refractive errors have been reported in several studies that used different ocular biometric methods [22,23,27,28,29]. Such errors may be attributable to AL inaccuracies but the use of old IOL power formulae may also play a role. Third-generation formulae are very dependent on kerato-metric and anterior chamber depth values. Any measurement inaccuracy of these parameters or a failure to use the most appropriate formula may affect the final refractive results [32,33]. Mono-focal IOL is the implant of choice when PPVc is performed. We think that the implant of advanced technology IOLs, such multifocal, extended depth of focus (EDOF), or toric IOLs along with PPV could increase the unpredictability of the refractive outcome mostly in macula-off cases, in which the accuracy of the preoperative biometry is less reliable. Add-on multifocal IOLs could be considered after satisfactory RRD anatomical and functional repair. In this regard, however, it is useful to bear in mind that the possible influence of the vitreous absence on photic phenomena has not yet been reported.

In terms of surgical complications, some studies [4,22,28] have reported that the rates of intraoperative complications associated with PPVc and PPVa were the same, but others [18,20,23] have found higher rates during PPVa. The most frequent complication observed is posterior capsule rupture, followed by zonular dialysis and IOL dislocation, reflecting the lack of vitreous support during phacoemulsification. Cataract extraction after vitrectomy can be challenging, particularly for myopic eyes. In such complicated cases, iris-claw fixation IOLs are commonly placed, but implant positioning can be affected by further pupil distortion or iris atrophy [11,12,34,35]. The most common postoperative complications are epiretinal membrane formation, macular edema, and elevated IOP; the frequencies tend to be similar after both procedures [4,22] or higher after PPVc [17,20,23,28]. Takahashi et al. [36] and Josifovska et al. [37] found that RRD per se increases the levels of intravitreal inflammatory cytokines compared to those of eyes with epiretinal membranes or macular holes. Addition of phacoemulsification to PPV may further increase the cytokine levels, especially when cataract surgery is performed by surgical fellows, possibly explaining the higher incidence of postoperative complications.

When PPV and cataract surgery are performed separately, the patient undergoes sedation or general anesthesia at least twice, and possibly three times if silicone oil removal is separately performed. This increases the risks associated with anesthesia, and costs (multiple hospital admissions, visits, medications, and professional fees). The only study that compared costs strongly supported PPVc compared to PPVa followed by cataract removal [26].

In conclusion, the primary success rate of RRD repair is generally comparable after PPVc and PPVa. The rate of intraoperative complications is lower for the combined procedure, but the rate of postoperative complications is higher. Macula-off RRD treated via PPVc is commonly associated with myopic refractive error; the ocular biometry method used and the IOL power calculation formula employed should be chosen with great care. Surgical experience is also important. We suggest that PPVa rather than PPVc should be particularly considered for younger patients with the aim of maintaining the accommodation function. In such patients, scleral buckling or the combination of PPV with an encircling band is also an effective option.

The English in this document has been checked by at least two professional editors, both native speakers of English. For a certificate, please see:

http://www.textcheck.com/certificate/o8BlxU (accessed on 20 July 2023)

## Figures and Tables

**Table 1:** Characteristics of the included studies.

First Author, Year	PPVc (N°)	PPVa (N°)	Follow Up (Months)	Design
Lee et al., 2012 [18]	311	54	>3	R
Seider et al., 2014 [26]	n.a.	n.a.	n.a.	R
Caiado et al., 2015 [19]	28	69 (41 pseudo-phakic)	>12	R
Kim et al., 2015 [27]	38	25	>1	R
Erçalık et al., 2017 [20]	458	376	>6	R
Loukovaara et al., 2018 [17]	126	1564	12	R
Guber et al., 2019 [21]	516	501	n.a.	R
Helmy et al., 2020 [28]	20	20	4	PR
Tan et al., 2021 [22]	127	139	>6	R
Mora et al., 2021 [4]	30	29	>6	PR
Moussa et al., 2021 [29]	70	41	3 to 6 after gas reabsorption or oil removal	R
Kim et al., 2021 [23]	82	111	>6 (mean 26.3)	R
Radeck et al., 2022 [30]	2163	451	3	R

PPVa: pars plana vitrectomy alone; PPVc: pars plana vitrectomy combined with cataract extraction, n.a.: not available; R = retrospective; PR = prospective randomized.

**Table 2:** Rate of RRD successful repair in the considered studies.

First Author, Year	PPVc (N°)	PPVa (N°)	Tamponade	SR of PPVc (%)	SR of PPVa (%)	*p* Value
Caiado et al., 2015 [19]	28	28 (phakic)	Gas, SO	91 with Gas 94 with SO	71 with Gas 72 with SO	0.043 *
Loukovaara et al., 2018 [17]	126	1564	Gas, SO	81	90.5	n.a.
Guber et al., 2019 [21]	516	501	Gas, SO	89.3	90.4	N.S.
Helmy et al., 2020 [28]	20	20	SO	100	99.5	N.S.
Tan et al., 2021 [22]	127	139	Gas	84.3	89.2	N.S.
Mora et al., 2021 [4]	30	29	Gas	96.7	89.7	N.S.
Kim et al., 2021 [23]	82	111	Air, Gas	91.5	92.8	N.S.
Radeck et al., 2022 [30]	2163	451	Gas, SO	93	88.7	0.002 *

PPVa: pars plana vitrectomy alone; PPVc: pars plana vitrectomy combined with cataract extraction; SO: silicone oil; SR: success rate; * statistically significant; N.S. not significant (>0.05); n.a.: not available.

**Table 3:** Postoperative refraction of the considered studies.

First Author, Year	PPVc (N°)	PPVa (N°)	Ocular Biometry	IOL Power Formula	ME PPVc (SE in D)	ME PPVa (SE in D)	*p* Value
Kim et al., 2015 [27]	38	25	IOL Master, ARK, ultrasound	SRK/t	−0.40 ± 1.07 D	0.07 ± 0.56 D	0.028 *
Tan et al., 2021 [22]	127	139	IOL Master, ARK, ultrasound	SRK/t	−0.32 ± 1.28 D	0.16 ± 1.53 D	0.047 *
Mora et al., 2021 [4]	30	29	IOL Master 500, ultrasound	SRK/t	n.a.	n.a.	n.a.
Moussa et al., 2021 [29]	70	41	IOL Master 700, ultrasound	Hoffer Q if AL < 22 mm SRK/t if AL > 22 mm	−0.08 ± 0.92 D	−0.07 ± 0.56 D	n.a
Kim et al., 2021 [23]	82	111	IOL Master, ultrasound	n.a.	−0.58 ± 0.97 D	−0.18 ± 0.84 D	0.014 *

PPVa: pars plana vitrectomy alone; PPVc: pars plana vitrectomy combined with cataract extraction; IOL: intraocular lens; ARK: autorefractor keratometer; ME: mean refractive error; SE: spherical equivalent, D: diopters, n.a.: not available. * statistically significant.

## Data Availability

All data and material are available from the corresponding author.
